# Inactivation of yellow fever virus by WHO-recommended hand rub formulations and surface disinfectants

**DOI:** 10.1371/journal.pntd.0012264

**Published:** 2024-06-20

**Authors:** Toni Luise Meister, Nicola Frericks, Robin D. V. Kleinert, Estefanía Rodríguez, Joerg Steinmann, Daniel Todt, Richard J. P. Brown, Eike Steinmann

**Affiliations:** 1 Department for Molecular and Medical Virology, Ruhr University Bochum, Bochum, Germany; 2 Institute for Infection Research and Vaccine Development (IIRVD), Centre for Internal Medicine, University Medical Centre Hamburg-Eppendorf, Hamburg, Germany; 3 Department for Clinical Immunology of Infectious Diseases, Bernhard Nocht Institute for Tropical Medicine (BNITM), Hamburg, Germany; 4 German Centre for Infection Research (DZIF), Partner site Hamburg-Lübeck-Borstel-Riems, Hamburg, Germany; 5 Division of Veterinary Medicine, Paul-Ehrlich-Institute, Langen, Germany; 6 Bernhard Nocht Institute for Tropical Medicine, Hamburg, Germany; 7 Institute of Clinical Hygiene, Medical Microbiology and Infectiology, General Hospital Nürnberg, Paracelsus Medical University, Nuremberg, Germany; 8 Institute of Medical Microbiology, University Hospital of Essen, Essen, Germany; 9 European Virus Bioinformatics Center (EVBC), Jena, Germany; 10 German Centre for Infection Research (DZIF), External Partner Site, Bochum, Germany; Faculty of Science, Ain Shams University (ASU), EGYPT

## Abstract

Despite continued outbreaks of yellow fever virus (YFV) in endemic regions, data on its environmental stability or guidelines for its effective inactivation is limited. Here, we evaluated the susceptibility of the YFV 17D vaccine strain to inactivation by ethanol, 2-propanol, World Health Organization (WHO)-recommended hand rub formulations I and II, as well as surface disinfectants. In addition, two pathogenic strains were tested to compare inactivation kinetics by WHO-recommended hand rub formulations I and II. Furthermore, environmental stability of the vaccine strain was assessed. YFV 17D particles displayed infectivity half-life decay profiles of ~13 days at room temperature. Despite this extended environmental stability, YFV was efficiently inactivated by alcohols, WHO-recommended hand formulations, and four out of five tested surface disinfectants. These results are useful in defining disinfection protocols to prevent non-vector borne YFV transmission.

## Author summary

Yellow fever virus (YFV) outbreaks persist in certain regions, emphasizing the need for effective inactivation methods. Our study focused on the YFV 17D vaccine strain, evaluating its susceptibility to commonly used disinfectants. Surprisingly, despite a prolonged half-life of ~13 days at room temperature, YFV proved susceptible to alcohols, WHO-recommended hand rubs, and most surface disinfectants tested. Consistent with these findings, WHO-recommended hand rubs effectively inactivated two pathogenic strains of YFV. This is essential information for establishing protocols to curb YFV transmission beyond vector-related scenarios. Our findings contribute to the broader understanding of YFV control, offering practical insights that can inform public health measures.

## Background

Yellow fever (YF) is a mosquito-borne viral illness caused by the yellow fever virus (YFV), an arbovirus from the genus *Flavivirus* within the family *Flaviviridae*. YFV circulates in an enzootic or jungle cycle between non-human primates and susceptible vectors species in Sub-Saharan Africa (*Aedes* sp. mosquitoes) and South American rain forests (*Sabethes* and *Haemagogus* sp. mosquitoes) [1]. In 2018, it was estimated that over 100,000 severe infections and 50,000 deaths occurred in Africa and South America [2]. Most symptomatic YFV infections in humans resolve within one week of onset, with patients experiencing fever, headache, chills, muscle pain, and nausea. However, in 10–25% of cases, infected individuals go on to further develop a hemorrhagic fever, characterized by pansystemic viral sepsis with viremia, high fever, kidney and liver damage, and a case-fatality rate of 20–50% [3]. Changes in the mode of transmission from a forest or jungle cycle to an urban cycle are characterized by a switch to human-to-human transmission, mediated by urban *Aedes aegypti* mosquitoes [4]. Historically, outbreaks were recorded as early as 1737 in the USA, with a major outbreak in Philadelphia in the summer of 1793 causing over 5000 deaths [5]. Although urban outbreaks have been mostly eliminated in South America, they continue to happen in Africa [6,7].

In December 2015, a yellow fever outbreak in Angola and the Republic of Congo quickly spread within densely populated urban areas, with international travel further facilitating spread to Kenya, the Democratic Republic of the Congo and China [8]. Consequently, this led the WHO in 2016 to classify this yellow fever epidemic as a global threat [9]. Indeed, the largest outbreak of the last 50 years took place in Brazil between 2016–2018, with 2,154 cases and 745 recorded deaths [10]. Despite no effective treatments, an effective live-attenuated YF vaccine has been available since the 1930s [1,11]. Unfortunately, vaccination programs are frequently hampered during YFV outbreaks in resource-limited countries due to limited vaccine supply.

Rare cases of non-vector mediated transmission in the pre-vaccine era were described, where scientists, clinicians and laboratory workers became infected with YFV after contact with blood or tissues of infected laboratory animals or when handling experimentally-infected animals [12,13]. Documenting and confirming non-vector routes of transmission are challenging as mosquitoes are ubiquitous in most countries with YFV and bites occur despite preventive measures. Therefore, vector-borne transmissions are implicated foremost as the main exposure. Non-vector transmissions are more likely to be suspected in non-endemic countries that detect unusual infections with links to returning traveller or unusual exposures [14].

As different countries and institutions have various procedures for YFV decontamination, no standardized guidelines for reducing risks of occupational exposure to YFV have been established to date. In this study, we tested the stability of YFV and its inactivation profile to various disinfection reagents.

## Methods

### Cell culture and virus propagation

BHK-21, Huh7, Huh7.5.1 and VeroE6 cells were propagated in DMEM supplemented with 10% FCS, 100 Units/mL penicillin, 100 μg/mL streptomycin and 2 mM L-glutamine at 37°C and 5% CO_2_. The YFV 17D vaccine strain (Stamaril^®^: Sanofi Pasteur) was kindly provided by Sandra Ciesek (Institute for Medical Virology, University Hospital, Goethe University, Frankfurt, Germany). YFV strains Uganda 1948 (UVE/YFV/1948/UG7MR896 TVP3236: 001V-02235) and Ivory Coast 1999 (IC99-01, Ref-SKU: 002V-02714) were obtained from the European Virus Archive Global (EVAg) and represent genetically distinct East African Lineage and West African Lineage strains respectively. For 17D and UG48 production, BHK-21 cells were seeded at 3×10^**6**^ cells/T75 flask in 10% FCS-containing DMEM. After 24 h the medium was changed to 5% FCS-containing DMEM with YFV (MOI 0.1) and incubated for 1 h at 37°C. Afterwards the medium was replaced with fresh 10% FCS containing DMEM and the cells were incubated for 4 days. Upon visible cytopathic effect, the supernatant was collected and centrifuged at 1,000 rpm for 5–10 min to remove any cell debris. YFV UG48 was further concentrated using Vivaspin 20 Centrifugal Concentrator tubes with a cutoff size of 100kDa. The supernatant was concentrated at 3000rcf for 15min to reduce the virus carrying volume to (at least) one third of the initial input. The IC99 strain was amplified similarly to 17D and UG48, but in VeroE6 cells. Viral titers were determined by plaque assay (YFV UG48 and IC99) or end-point dilution assay (YFV 17D). All viruses were aliquoted and stored at -80°C until further usage. Experiments using the vaccine strain YFV 17D were carried out in biosafety level (BSL) 2 laboratories in the Department for Molecular and Medical Virology at Ruhr University Bochum. YFV UG48 and IC99 were handled under BSL 3 conditions in the Division of Veterinary Medicine at Paul Ehrlich Institute, Langen, and at the Bernhard Nocht Institute for Tropical Medicine, Hamburg, respectively.

### YFV stability testing

Nine parts YFV 17D and one-part interfering substance (BSA, final concentration 0.3 g/L, clean condition) were combined in a tube according to our previous study [15]. The virus solutions were stored at room temperature (RT: 21°C) and at 4°C. Remaining infectious virus was determined at different time points (0, 0.2, 0.5, 1, 2, 3, 5, 7, 9, 12, 15, 22, 30 and 43 days) by an endpoint dilution assay on Huh7 cells. Therefore, Huh7 cells were seeded one day prior titration on a 96 well plate at 1×10^4^ cells/well. At the mentioned time points, the virus solution was added to the first row of the cells and serially diluted eight times. After six days at 37°C with 5% CO_2_, the cytopathic effect was evaluated by crystal violet staining. Remaining infectious viral titers (50% tissue culture infectious dose [TCID_50_/mL]) were calculated according to Spearman and Kärber. For each timepoint three independent replicates were collected.

### YFV inactivation by WHO-recommended hand rub formulations, ethanol, and 2-propanol

Virucidal activity of WHO-recommended hand rub formulation I and II (Table 1), as well as ethanol, and 2-propanol were assessed based on European guideline EN14476, as described previously [16]. Specifically, eight parts of disinfectant (WHO I, WHO II, ethanol and 2-propanol) or cell culture medium for the untreated control (UTC) were mixed with one-part interfering substance (bovine serum albumin [BSA], final concentration 0.3 g/L, clean condition) and one-part YFV 17D, UG48 or IC199 and incubated for 30 s at RT (21°C). An end-point dilution assay was performed on Huh7 (17D), Huh7.5.1 (UG48) or VeroE6 (IC99) cells to determine remaining infectious viral titers as described above. Cell viability was assessed assessed following a similar protocol using one-part of 1×PBS instead of virus. WHO formulation I and II as well as ethanol and 2-propanol were tested at final concentrations of 20%, 30%, 40%, 60% and 80%. The experiment was performed in three independent replicates.

**Table 1:** WHO-recommended hand rub formulations.

Product	Ingredients	Concentration	Incubation time
ethanol	≥99,8% ethanol	20, 30, 40, 60 and 80%	30 s
2-propanol	≥99,8% 2-propanol	20, 30, 40, 60 and 80%	30 s
WHO formulation I	80% ethanol (v/v) 1.45% glycerol (v/v)0.125% hydrogen peroxide (v/v)	20, 30, 40, 60 and 80%	30 s
WHO formulation II	75% 2-propanol (v/v) 1.45% glycerol (v/v) 0.125% hydrogen peroxide (v/v)	20, 30, 40, 60 and 80%	30 s

### YFV inactivation by surface disinfectants

Stainless steel discs (2 cm diameter discs, article no. 4174–3000, GK Formblech GmbH, Berlin, Germany) were decontaminated in 70% (v/v) ethanol for 15 min. Subsequently, the stainless-steel discs were contaminated with 50 μL virus solution containing nine parts YFV 17D and one-part interfering substance (BSA, final concentration 0.3 g/L, clean condition). The steel discs were incubated at RT (21°C) until the virus solution was desiccated completely. Subsequently, 100 μL of surface disinfectant (Table 2) at the indicated concentrations was applied onto the stainless-steel disc and incubated according to the manufacturer’s instructions. Cell culture medium was used for the untreated control (UTC). Hereafter, the contaminated stainless steel discs were transferred into a 25 mL container harboring 2 mL cell culture medium (without FCS) and subsequently vortexed. To determine viral titers an end-point dilution assay was performed on Huh7 cells as described above. The experiment was performed in three independent replicates.

**Table 2:** Surface disinfectants.

Product	Ingredients	Concentration	Incubation time
Bacillol AF	450 mg/g 1-propanol, 250 mg/g 2-propanol, 47 mg/g ethanol	80%	30 s
Antifect N liquid	250 mg/g ethanol, 350 mg/g 2-propanol	80%	30 s
Kohrsolin FF	50 mg/g glutaraldehyde, 30 mg/g benzylalkyldimethyl-ammonium chloride, 30 mg/g didecyldimethyl-ammonium chloride	0.5%	5 min
Incidin Rapid	98 mg/g glutaraldehyde, 50 mg/g alkyldimethylbenzyl-ammonium chloride, 50 mg/g didecyldimethyl-ammonium chloride	0.5%	5 min
Incidin OxyFoam	15 mg/g hydrogen peroxide	80%	30 s

### Statistical analysis

Reduction factors (RFs) were calculated by subtracting the residual viral titers from the titer of the UTC. Reduction factors are displayed in logTCID_50_.

Inactivation kinetics of YFV by WHO formulation I and II was compared to other viruses such as Zika virus, Ebola virus and Chikungunya virus as well as the reference virus MVA (Modified Vaccinia Ankara) using robust Hill non-linear dose-response fit.

## Results

**Fig 1:**
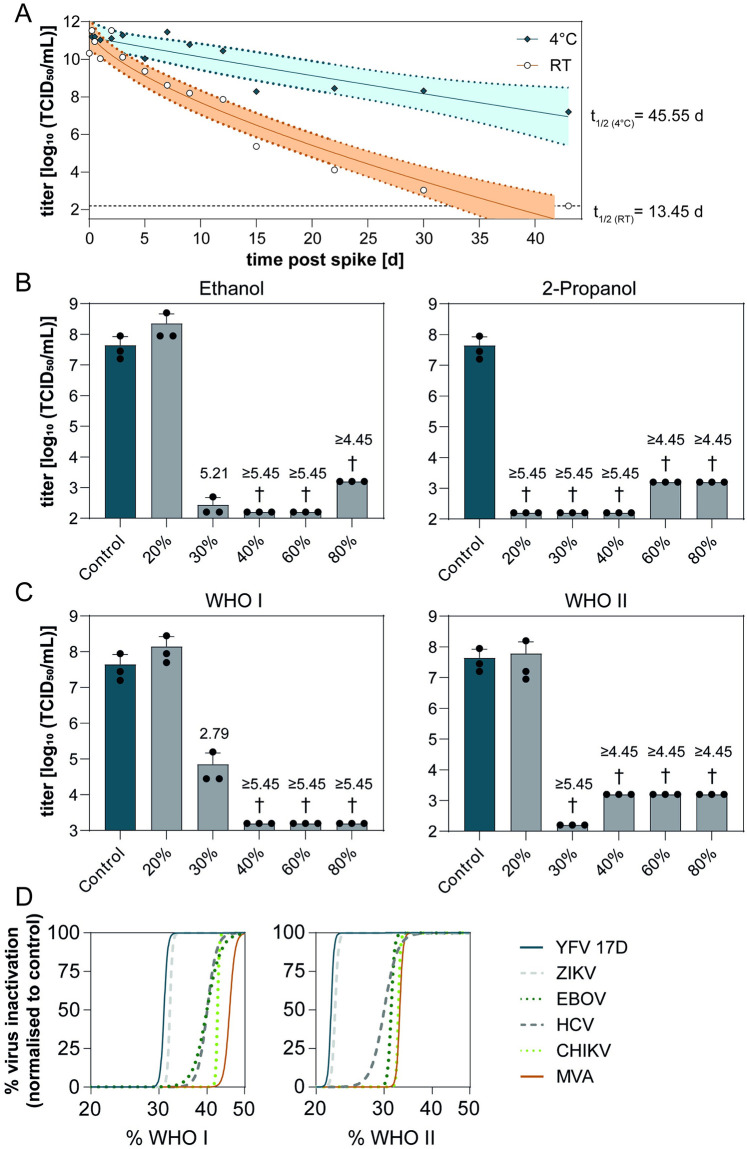
**(A) Stability of YFV 17D**. Nine parts YFV 17D were mixed with one-part interfering substance (BSA, 0.3 g/L final concentration) and incubated for the indicated time periods at either room temperature (21°C) or 4°C. Remaining infectious viral titers were recovered from the contaminated stainless steel discs at the indicated time points. Viral titers were determined by an end point dilution assay and calculated as TCID_50_/mL. A non-linear model based on a Weibull distribution was used to fit data. Dotted black line indicates the lower limit of detection. Colored area displays 95% confidence interval. **(B and C) Inactivation of YFV 17D by WHO-recommended hand rub formulations and ethanol or 2-propanol**. All four disinfectants were tested regarding their potential to inactivate YFV 17D in a quantitative suspension test according to EN14476. Ethanol and 2-propanol (B) as well as WHO formulation I and II (C) were diluted to 20, 30, 40, 60 and 80% final concentration and mixed with one-part interfering substance (BSA, 0.3 g/L final concentration) and one-part YFV 17D for 30 s. Remaining infectious viral titers were determined in an end-point dilution assay and are displayed as TCID_50_/mL. The untreated control (UTC) is displayed as the dark blue bar. The light grey bar shows viral titers recovered after exposure to the disinfectants. The cross (†) indicates a reduction of infectious viral titers to the lower limit of detection. Numbers above the grey bars indicate reduction factors (RFs) compared to the UTC. Each dot indicates one biological replicate. All experiments were performed three times. **(D) Inactivation by WHO formulation I and II**. Inactivation kinetics were compared to other viruses such as ZIKV (Zika virus), EBOV (Ebola virus), HCV (Hepatitis C virus), CHIKV (Chikungunya virus) as well as the reference virus MVA (Modified Vaccinia Ankara) using robust non-linear fitting.

To evaluate YFV stability at different temperatures, virus suspensions were incubated at either 4°C or 21°C for a total of 43 days. When incubated at RT (21°C), YFV infectivity was reduced by one order of magnitude after ~5 days and further declined to 1×10^6^ TCID_50_/mL after ~15 days with a half-life of ~13 days (Fig 1A). Viral infectivity at 4°C decreased moderately and maintained high titers (1×10^8^ TCID_50_/ml) with half-life times of ~45 days after the incubation period (Fig 1A). The time required for the absence of any detectable infectious virus was estimated to be ~37 days (95% CI = 30.02–43.53 days) at RT and ~95 days (95% CI = 75.33–151.01 days) at 4°C.

Alcohols are the active ingredients of commercial alcohol-based antiseptics and disinfectants used in medical settings for the decontamination of solutions, skin or wounds that were exposed to potential viruses. These hand disinfection products or reagents are tested in so-called suspension tests to evaluate the performance of the disinfectant in terms of concentration and contact time ratio in a liquid environment. To evaluate the susceptibility of YFV 17D to inactivation by alcohols, we exposed the virus to increasing concentration of ethanol or 1-propanol at 20%, 30%, 40%, 60% and 80% final concentration in a quantitative suspension test according to EN14476. Both ethanol and 2-propanol (Fig 1B) reduced viral titers to background levels at concentrations >40% and >20% (vol/vol), respectively. WHO-recommended hand rub formulations have proven effective against a variety of viruses. Therefore, we analysed the virucidal efficacy of WHO formulations I and II (Table 1) using the same procedure. The WHO formulation II, a propanol-based disinfectant, exerted a stronger antiviral effect than the ethanol-based WHO formulation I (Fig 1C). Similar results were obtained for two pathogenic YFV strains (S1 Fig). Interestingly, YFV 17D displayed a lower degree of resistance to inactivation by the WHO formulations I and II than other currently emerging viruses (Fig 1D) including Ebola virus (EBOV) and Zika virus (ZIKV) [17] as well as Modified Vaccinia virus Ankara (MVA) which serves as a gold standard for enveloped viruses in chemical inactivation assays [18].

**Fig 2:**
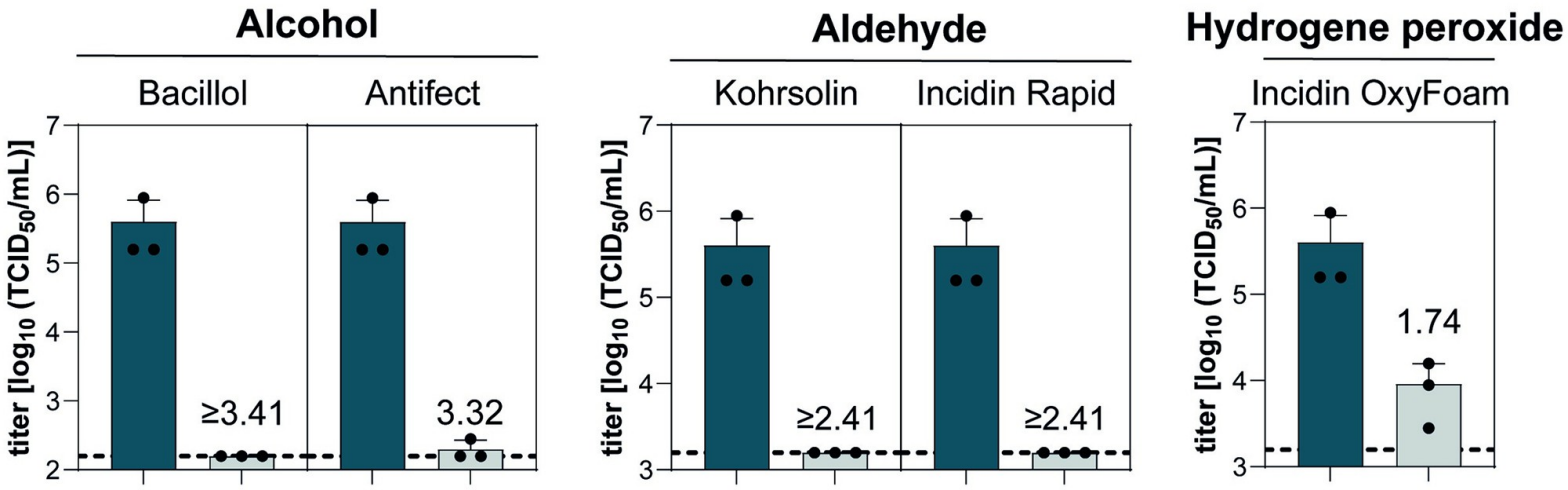
Inactivation of YFV 17D by surface disinfectants. Five surface disinfectants based on alcohol, aldehyde, and hydrogen peroxide were tested according to EN16777 to evaluate their virucidal activity. All products were tested as suggested by the manufacturers. Nine parts YFV 17D mixed with one-part interfering substance (BSA, 0.3 g/L final concentration) were spiked and dried on sterile stainless steel discs. Subsequently the virus was inactivated with undiluted Bacillol AF, Antifect N liquid and Incidin OxyFoam for 30 s. Kohrsolin FF and Incidin Rapid were diluted to 0.5% working solutions and incubated for 5 min on contaminated stainless steel discs. Remaining infectious viral titers were determined in an end-point dilution assay and are displayed as TCID_50_/mL. The untreated control (UTC) is displayed as the dark blue bar. The light grey bar shows viral titers recovered after exposure to the surface disinfectants. Dotted black line indicates the lower limit of detection. Numbers above the grey bars indicate reduction factors (RFs) compared to the UTC. Each dot indicates one biological replicate. The experiment was performed in three independent replicates.

Viruses may persist on surfaces for several days, or even months, and can be transferred directly from contaminated surfaces to susceptible individuals. To simulate surface disinfection, we established an experimental system to test the stability on inanimate surfaces. Stainless-steel discs providing a smooth surface were used on which the virus suspension was applied. After the virus suspension has dried, the ability of disinfectants to inactivate potentially residual infectivity was monitored. The dried virus suspension was covered with five different commercial surface disinfectants (Table 2) and their virucidal activities were determined by an end-point dilution assay. The alcohol- and aldehyde-based surface disinfectants inactivated YFV to background levels, whereas the hydrogen peroxide product minimally reduced viral infectivity with a reduction factor of 1.7 (Fig 2).

## Discussion

In this study, we performed the first comprehensive profiling of YFV environmental stability and susceptibility to chemical inactivation, to inform risk assessments aimed at minimizing non-vector borne transmission of YFV in laboratory and hospital settings. Overall, non-vector borne infections of humans for YFV are rare but have not been systematically evaluated, and only single case reports have been described. For instance, the vaccine strain of YFV has been transmitted via breastmilk resulting in febrile illness and meningoencephalitis in infants [14,19,20]. Therefore, information about preventive hygiene measures are important and also relevant for other related members of the genus *Flavivirus* [14]. Analogous to mosquito-borne infection, non-vector transmission depends on several parameters including the titer of the virus inoculum and the volume of material that reaches potential sites of infection [21]. Additional factors that influence the likelihood of direct transmission include environmental stability of the virus, the immune status of individuals potentially exposed, and the route of virus contact and entry [22]. Here, we demonstrated extended YFV stability in a liquid environment, with infectious virus still detectable after 37 days incubation at RT. At 4°C, this environmental stability was extended, with high viral titers still observed after the incubation period. These experiments were conducted with BSA as protein carrier to mimic blood contamination in real-world scenarios and allow comparisons between different laboratories. In comparison to HCV probed in the same assay setup [23], YFV demonstrates longer half-life times. Several reports described nosocomial transmissions [21] due to flavivirus-positive blood exposure or virus-contaminated cerebrospinal fluid (CSF), urine, or other fluids to which clinicians and laboratory workers are regularly exposed to. Therefore, guidelines for effective disinfection procedures are important to prevent such transmission routes. When testing ethanol and 2-propanol as active ingredients of commercial alcohol-based antiseptics and disinfectants used in medical settings, YFV was effectively inactivated at low concentrations. In the field of pathogen reduction in blood products, it has been shown that the Amotosalen/ultraviolet A (UVA) light technology was able to inactivate YFV in human platelets and plasma [24]. The WHO proposed in its 2009 *Guidelines on Hand Hygiene in Health Care* the use of 2 alcohol-based hand rubs (formulation I and formulation II) for surgical and hygiene hand disinfection in healthcare settings to reduce the transmission of pathogens by hands [17,25]. We and others have recently shown that severe acute respiratory syndrome coronavirus 2 (SARS-CoV-2), middle east respiratory syndrome coronavirus (MERS-CoV), SARS, Mpox virus (MPXV), and other viruses were also inactivated by these hand-rub formulations and alcohols [15]. These findings were replicated for YFV, which was highly susceptible to inactivation when exposed to WHO formulations I and II compared to other emerging viruses, with a comparable profile to the closely related ZIKV. Five different surface disinfectants based on alcohol, aldehyde and hydrogen peroxide were tested regarding their potential to inactivate infectious YFV. All surface disinfectants reduced viral titers to background levels except the hydrogen peroxide–based product. Similar findings have recently been published for MPXV [26]. In a study by Ajorio et al., hydrogen peroxide concentrations of at least 10% for 20 min exposure time were required to inactivate YFV for vaccine usage [27]. It should be considered that in our study, only stainless-steel discs were used to test surface disinfectants and future studies should also examine YFV stability on other materials. Nonetheless, our findings reveal that YFV was efficiently inactivated by alcohols and WHO recommended formulations validating their utility in the context of the healthcare system and YFV outbreak situations.

## Supporting information

S1 Fig The two disinfectants were tested regarding their potential to inactivate YFV in a quantitative suspension test according to EN14476. WHO formulation I and II (C) were diluted to 20, 30, 40, 60 and 80% final concentration and mixed with one part interfering substance (BSA, 0.3 g/L final concentration) and one part YFV for 30 s. Remaining infectious viral titers were determined in an end-point dilution assay and are displayed as TCID50/mL. The untreated control (UTC) is displayed as the dark blue bar. The light grey bar shows viral titers recovered after exposure to the disinfectants. The cross (†) indicates a reduction of infectious viral titers to the lower limit of detection. Numbers above the grey bars indicate reduction factors (RFs) compared to the UTC. Each dot indicates one biological replicate. All experiments were performed three times.
(DOCX)

S1 Supplementary Information Includes raw data tables. (DOCX)
