# Supplementary material for: Inactivation of yellow fever virus by WHO-recommended hand rub formulations and surface disinfectants
Source: PLoS Negl Trop Dis. 2024 Jun 20;18(6):e0012264. doi: 10.1371/journal.pntd.0012264 (PMC11218936; doi:10.1371/journal.pntd.0012264)
Supplement: S1 Fig — WHO formulation I and II (C) were diluted to 20, 30, 40, 60 and 80% final concentration and mixed with one part interfering substance (BSA, 0.3 g/L final concentration) and one part YFV for 30 s. Remaining infectious viral titers were determined in an end-point dilution assay and are displayed as TCID50/mL. The untreated control (UTC) is displayed as the dark blue bar. The light grey bar shows viral titers recovered after exposure to the disinfectants. The cross (†) indicates a reduction of infectious viral titers to the lower limit of detection. Numbers above the grey bars indicate reduction factors (RFs) compared to the UTC. Each dot indicates one biological replicate. All experiments were performed three times. (DOCX) [file pntd.0012264.s001.docx]

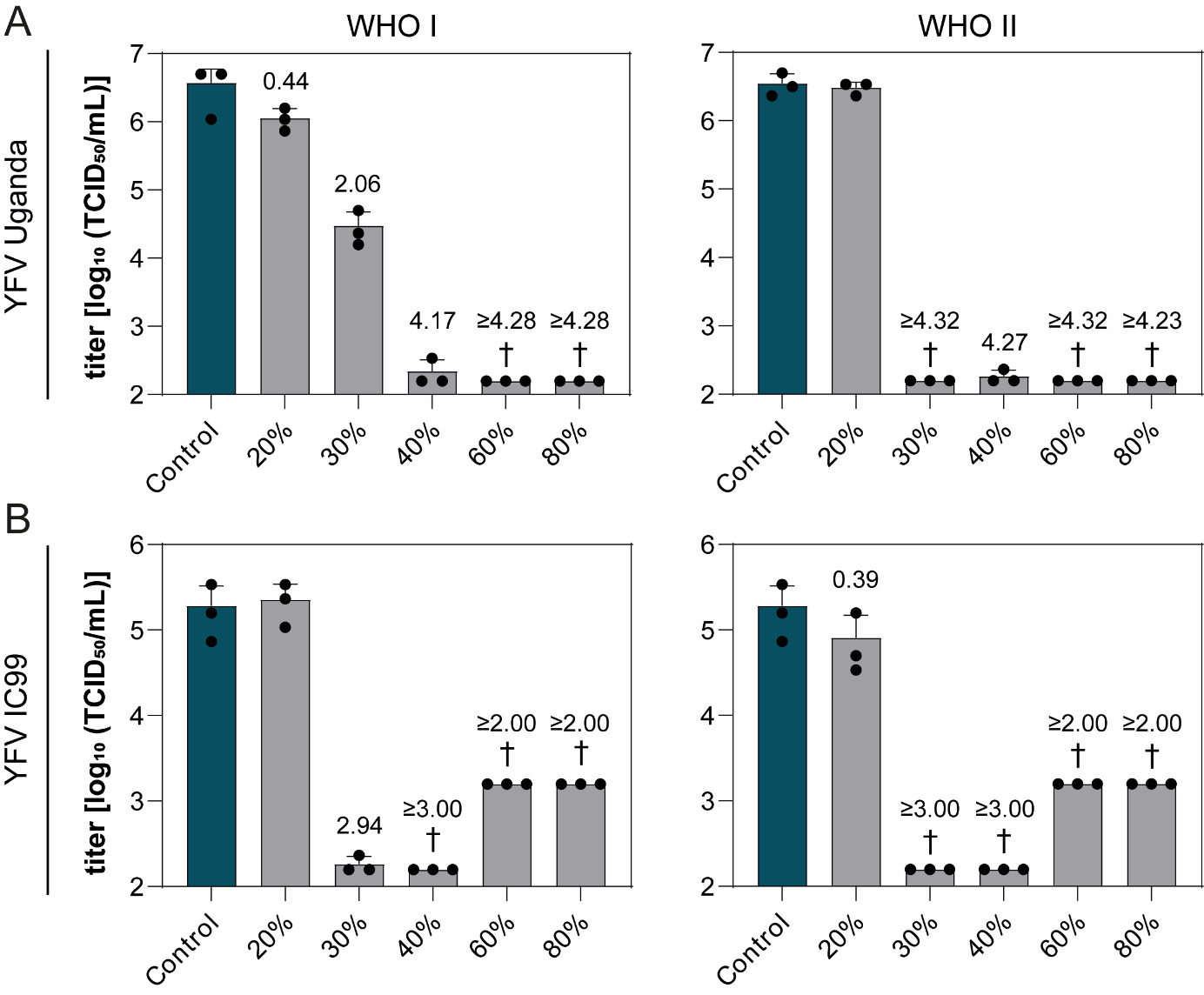


**S1 Fig. Inactivation of YFV Uganda and IC99 by WHO-recommended hand rub formulations.** The two disinfectants were tested regarding their potential to inactivate YFV in a quantitative suspension test according to EN14476. WHO formulation I and II (C) were diluted to 20, 30, 40, 60 and 80% final concentration and mixed with one part interfering substance (BSA, 0.3 g/L final concentration) and one part YFV for 30 s. Remaining infectious viral titers were determined in an end-point dilution assay and are displayed as TCID_50_/mL. The untreated control (UTC) is displayed as the dark blue bar. The light grey bar shows viral titers recovered after exposure to the disinfectants. The cross (†) indicates a reduction of infectious viral titers to the lower limit of detection. Numbers above the grey bars indicate reduction factors (RFs) compared to the UTC**.** Each dot indicates one biological replicate. All experiments were performed three times.
