# Supplementary material for: Inactivation of yellow fever virus by WHO-recommended hand rub formulations and surface disinfectants
Source: PLoS Negl Trop Dis. 2024 Jun 20;18(6):e0012264. doi: 10.1371/journal.pntd.0012264 (PMC11218936; doi:10.1371/journal.pntd.0012264)
Supplement: S1 Supplementary Information — (DOCX) [file pntd.0012264.s002.docx]

**S1 Supplementary Information**

**Inactivation of Yellow Fever Virus by WHO-Recommended Hand Rub Formulations and surface disinfectants**

Toni Luise Meister^1,2,3,4^, Nicola Frericks^1^, Robin D. V. Kleinert^5^, Estefanía Rodríguez^4,6^, Joerg Steinmann^7,8^, Daniel Todt^1,9^, Richard J. P. Brown^1,5*^, and Eike Steinmann^1,10*^

Table 1: YFV 17D stability in solution at different temperatures. Mean values of three independent replicates. Titres are depicted in logTCID_50_/mL. Data belongs to Fig 1A. * indicates reduction of viral titres to background levels.

| days | RT | | | 4 °C | | |
| --- | --- | --- | --- | --- | --- | --- |
| 0 | 9.95 | 10.70 |  | 9.95 | 10.70 |  |
| 0.2 | 11.45 | 11.95 | 11.20 | 9.95 | 11.95 | 11.70 |
| 0.5 | 10.70 | 10.70 | 11.45 | 10.95 | 11.45 | 11.20 |
| 1 | 9.70 | 9.70 | 10.70 | 11.45 | 9.70 | 11.95 |
| 2 | 11.45 | 11.70 | 11.45 | 9.70 | 12.20 | 11.45 |
| 3 | 8.95 | 10.70 | 10.70 | 9.70 | 11.45 | 12.70 |
| 5 | 9.95 | 7.95 | 10.20 | 10.70 | 9.20 | 10.20 |
| 7 | 8.45 | 8.70 | 8.70 | 10.45 | 10.95 | 12.95 |
| 9 | 8.70 | 7.70 |  | 9.95 | 12.70 | 9.70 |
| 12 | 8.20 | 6.95 | 8.45 | 10.20 | 11.20 | 9.95 |
| 15 | 6.20 | 3.95 | 5.95 | 9.20 | 7.45 | 8.20 |
| 22 | 4.95 | 2.45 | 4.95 | 8.45 | 8.70 | 8.20 |
| 30 | 3.20 | 2.20* | 3.70 | 7.95 | 8.70 |  |
| 43 | 2.20* | 2.20* | 2.20* | 7.20 | 5.95 | 8.45 |

Table 2: YFV 17D inactivation by different concentrations of ethanol. Titres are depicted in logTCID_50_/mL. Data belongs to Fig 1B. * indicates reduction of viral titres to background levels.

|  | Ethanol | | | | |
| --- | --- | --- | --- | --- | --- |
| Control | 20% | 30% | 40% | 60% | 80% |
| 7.95 | 8.70 | 2.20* | 2.20* | 2.20* | 3.20* |
| 7.20 | 7.95 | 2.70 | 2.20* | 2.20* | 3.20* |
| 7.45 | 7.95 | 2.20* | 2.20* | 2.20* | 3.20* |

Table 3: YFV 17D inactivation by different concentrations of 2-propanol. Titres are depicted in logTCID_50_/mL. Data belongs to Fig 1B. * indicates reduction of viral titres to background levels.

|  | 2-Propanol | | | | |
| --- | --- | --- | --- | --- | --- |
| Control | 20% | 30% | 40% | 60% | 80% |
| 7.95 | 2.20* | 2.20* | 2.20* | 3.20* | 3.20* |
| 7.20 | 2.20* | 2.20* | 2.20* | 3.20* | 3.20* |
| 7.45 | 2.20* | 2.20* | 2.20* | 3.20* | 3.20* |

Table 4: YFV 17D inactivation by different concentrations of WHO-recommended hand rub formulation I. Titres are depicted in logTCID_50_/mL. Data belongs to Fig 1C. * indicates reduction of viral titres to background levels.

|  | WHO-recommended hand rub formulation I | | | | |
| --- | --- | --- | --- | --- | --- |
| Control | 20% | 30% | 40% | 60% | 80% |
| 7.95 | 8.45 | 5.20 | 3.20* | 3.20* | 3.20* |
| 7.20 | 7.70 | 4.45 | 3.20* | 3.20* | 3.20* |
| 7.45 | 7.95 | 4.45 | 3.20* | 3.20* | 3.20* |

Table 5: YFV 17D inactivation by different concentrations of WHO-recommended hand rub formulation II. Titres are depicted in logTCID_50_/mL. Data belongs to Fig 1C. * indicates reduction of viral titres to background levels.

|  | WHO-recommended hand rub formulation II | | | | |
| --- | --- | --- | --- | --- | --- |
| Control | 20% | 30% | 40% | 60% | 80% |
| 7.95 | 8.20 | 2.20* | 3.20* | 3.20* | 3.20* |
| 7.20 | 6.95 | 2.20* | 3.20* | 3.20* | 3.20* |
| 7.45 | 7.20 | 2.20* | 3.20* | 3.20* | 3.20* |

Table 6: YFV 17D inactivation by different surface disinfectants. Titres are depicted in logTCID_50_/mL. Data belongs to Fig 2. * indicates reduction of viral titres to background levels.

|  | Alcohol | | Aldehyde | | Hydrogene peroxide | |
| --- | --- | --- | --- | --- | --- | --- |
| Control | Bacillol | Antifect | Kohrsolin | Incidin Rapid | Incidin Oxyfoam | |
| 5.20 | 2.20* | 2.45 | 3.20* | 3.20* | 3.45 |  |
| 5.20 | 2.20* | 2.20* | 3.20* | 3.20* | 3.95 |  |
| 5.95 | 2.20* | 2.20* | 3.20* | 3.20* | 4.20 |  |

Table 7: YFV Uganda inactivation by different concentrations of WHO-recommended hand rub formulation I. Titres are depicted in logTCID_50_/mL. Data belongs to S1A Fig. * indicates reduction of viral titres to background levels.

|  | WHO-recommended hand rub formulation I | | | | |
| --- | --- | --- | --- | --- | --- |
| Control | 20% | 30% | 40% | 60% | 80% |
| 6.03 | 5.87 | 4.37 | 2.20* | 2.20* | 2.20* |
| 6.70 | 6.20 | 4.20 | 2.20* | 2.20* | 2.20* |
| 6.70 | 6.03 | 4.70 | 2.53 | 2.20* | 2.20* |

Table 8: YFV Uganda inactivation by different concentrations of WHO-recommended hand rub formulation II. Titres are depicted in logTCID_50_/mL. Data belongs to S1A Fig. * indicates reduction of viral titres to background levels.

|  | WHO-recommended hand rub formulation II | | | | |
| --- | --- | --- | --- | --- | --- |
| Control | 20% | 30% | 40% | 60% | 80% |
| 6.70 | 6.37 | 2.20* | 2.20* | 2.20* | 2.20* |
| 6.37 | 6.53 | 2.20* | 2.20* | 2.20* | 2.20* |
| 6.50 | 6.53 | 2.20* | 2.37 | 2.20* | 2.20* |

Table 9: YFV IC99 inactivation by different concentrations of WHO-recommended hand rub formulation I. Titres are depicted in logTCID_50_/mL. Data belongs to S1B Fig. * indicates reduction of viral titres to background levels.

|  | WHO-recommended hand rub formulation I | | | | |
| --- | --- | --- | --- | --- | --- |
| Control | 20% | 30% | 40% | 60% | 80% |
| 4.87 | 5.53 | 2.37 | 2.20* | 3.20* | 3.20* |
| 5.20 | 5.37 | 2.20* | 2.20* | 3.20* | 3.20* |
| 5.53 | 5.03 | 2.20* | 2.20* | 3.20* | 3.20* |

Table 10: YFV IC99 inactivation by different concentrations of WHO-recommended hand rub formulation II. Titres are depicted in logTCID_50_/mL. Data belongs to S1B Fig. * indicates reduction of viral titres to background levels.

|  | WHO-recommended hand rub formulation II | | | | |
| --- | --- | --- | --- | --- | --- |
| Control | 20% | 30% | 40% | 60% | 80% |
| 4.87 | 4.53 | 2.20* | 2.20* | 3.20* | 3.20* |
| 5.20 | 5.20 | 2.20* | 2.20* | 3.20* | 3.20* |
| 5.53 | 4.70 | 2.20* | 2.20* | 3.20* | 3.20* |
